# 10.4 DIFFUSION WEIGHTED SPECTROSCOPY STUDIES OF CELL-TYPE SPECIFIC ABNORMALITIES IN SCHIZOPHRENIA

**DOI:** 10.1093/schbul/sby014.037

**Published:** 2018-04-01

**Authors:** Dost Ongur

**Affiliations:** McLean Hospital

## Abstract

**Background:**

In previous work we used diffusion tensor spectroscopy (DTS) to identify abnormal diffusion of the neuron-specific metabolite NAA in frontal white matter in patients with chronic schizophrenia in the absence of abnormalities in the diffusion of cell-type non-specific metabolites Cr and Cho.

**Methods:**

DTS relies on the same principles as DTI, but the diffusion characteristics of metabolites are probed, instead of those of water. Since brain metabolites are concentrated in specific cellular and sub-cellular compartments, their diffusion reflects the local geometry of these compartments. We have implemented a DTS approach at a 4 Tesla Varian MRI scanner (described in Du et al 2013).

**Results:**

We have now collected similar data from first episode psychosis patients and matched healthy controls. We find that NAA diffusion is normal in the frontal PFC in first episode patients, but Cr and Cho diffusion is abnormal.

**Discussion:**

Taken together, our studies suggest white matter abnormalities in non-neuronal elements in early phases of schizophrenia which are followed by neuronal damage in chronic disease.

